# A Full Evaporation Static Headspace Gas Chromatography Method with Nitrogen Phosphorous Detection for Ultrasensitive Analysis of Semi-volatile Nitrosamines in Pharmaceutical Products

**DOI:** 10.1208/s12248-021-00669-8

**Published:** 2022-01-06

**Authors:** Jinjian Zheng, Christine L. Kirkpatrick, Daniel Lee, Xinxin Han, Ana I. Martinez, Kimberly Gallagher, Rebecca K. Evans, Sanjay V. Mudur, Xihui Liang, Jennifer Drake, Leah A. Buhler, Mark D. Mowery

**Affiliations:** Analytical Chemistry in Development and Supply, Merck Manufacturing Division, Merck & Co., Inc., 126 E. Lincoln Ave, Rahway, New Jersey 07065 USA

**Keywords:** Full evaporation headspace sampling, Gas chromatography with nitrogen phosphorous detection, NDMA, Nitrosamine, Nitrosation inhibition

## Abstract

The recent detection of potent carcinogenic nitrosamine impurities in several human medicines has triggered product recalls and interrupted the supply of critical medications for hundreds of millions of patients, illuminating the need for increased testing of nitrosamines in pharmaceutical products. However, the development of analytical methods for nitrosamine detection is challenging due to high sensitivity requirements, complex matrices, and the large number and variety of samples requiring testing. Herein, we report an analytical method for the analysis of a common nitrosamine, N-nitrosodimethylamine (NDMA), in pharmaceutical products using full evaporation static headspace gas chromatography with nitrogen phosphorous detection (FE-SHSGC-NPD). This method is sensitive, specific, accurate, and precise and has the potential to serve as a universal method for testing all semi-volatile nitrosamines across different drug products. Through elimination of the detrimental headspace-liquid partition, a quantitation limit of 0.25 ppb is achieved for NDMA, a significant improvement upon traditional LC-MS methods. The extraction of nitrosamines directly from solid sample not only simplifies the sample preparation procedure but also enables the method to be used for different products as is or with minor modifications, as demonstrated by the analysis of NDMA in 10+ pharmaceutical products. The *in situ* nitrosation that is commonly observed in GC methods for nitrosamine analysis was completely inhibited by the addition of a small volume solvent containing pyrogallol, phosphoric acid, and isopropanol. Employing simple procedures and low-cost instrumentation, this method can be implemented in any analytical laboratory for routine nitrosamine analysis, ensuring patient safety and uninterrupted supply of critical medications.

**Supplementary Information:**

The online version contains supplementary material available at 10.1208/s12248-021-00669-8.

**Figure:**
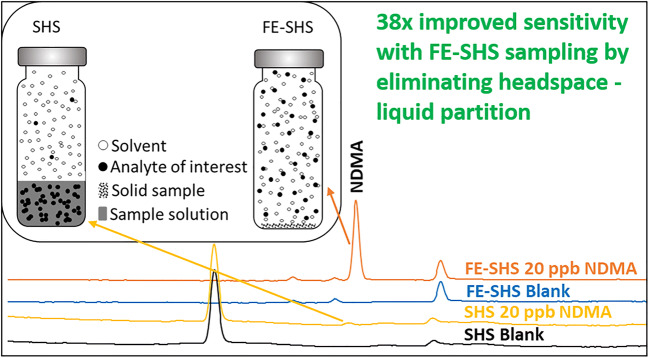
Graphical Abstract

## INTRODUCTION

The discovery of N-nitrosodimethylamine (NDMA), a potent probable carcinogen with very low acceptable intake (AI) limits (e.g., 96 ng/day), in valsartan has triggered industrywide scrutinization of nitrosamine contamination for all pharmaceutical products (1, 2). Since then, several drug products including sartans for hypertension, (3) metformin for diabetes (4), and ranitidine for heartburn (gastroesophageal reflux disease) (5) have been recalled due to unacceptable levels of NDMA. As new regulatory guidelines for nitrosamine risk require frequent testing in a broad range of pharmaceutical products, a fast, sensitive, and versatile analytical method is required to maintain supply. There are three major challenges in nitrosamine testing: (1) the array of products, (2) large number of batches to be tested, and (3) the high method sensitivity needed to meet regulatory expectations. There are approximately 1.13 billion people with hypertension (6) and over 463 million people with diabetes worldwide (7), and heartburn occurs in roughly 1.5 billion people on a weekly basis (8). Due to the widespread nature of these health issues, there are hundreds of thousands of batches of these drug products on the market. In addition, pharmaceutical companies need to perform confirmatory testing across multiple lots if a potential nitrosamine risk is identified in the drug product, which could affect a large portion of the product on the market or in development (9, 10). Furthermore, this is a rapidly evolving regulatory environment with major regulatory agencies actively revising guidance on the control of nitrosamines in human drugs. According to recent EMA guidance, drug manufacturers are required to demonstrate that the nitrosamine level is consistently below 10% of AI to justify omission of a specification (e.g., 4.8 ppb NDMA in metformin HCl based on a maximum daily dose of 2 g) (9). This is especially difficult for formulated drug products as extracting nitrosamines from complex matrices is much more difficult compared to drug substances.

Typically, nitrosamines are separated from sample matrix using liquid chromatography (LC) or gas chromatography (GC) and detected by a highly sensitive and specific detector. LC coupled with high resolution mass spectrometry (HRMS) is often used to achieve the desired sensitivity and selectivity (11–13). However, LC-HRMS is not widely available for routine use due to high instrument cost, high maintenance cost, and extensive analyst training. In addition, extensive sample preparation is required to minimize contamination to MS instrumentation, which limits the throughput. Although sensitive detection of nitrosamines using luminol chemiluminescence detection has been reported, the instrumentation is not commercially available (14). GC coupled with mass spectrometry (MS)(15), thermal energy analyzer (TEA)(16), nitrogen chemiluminescence detector (NCD), or nitrogen phosphorous detector (NPD)(17) have been used to analyze nitrosamines at ng/mL level. However, the challenge with these GC methods often lies in how to effectively extract nitrosamines from the complex sample matrix and how to prevent *in situ* formation of nitrosamines (18). These approaches often lead to the development of specific LC- or GC-MS methods for each product, which is time-consuming due to the large array of pharmaceutical products. So far, testing for nitrosamines using MS-based methods is mainly performed in a specialized lab equipped with mass spectrometers qualified for GMP testing and specialized analysts trained in their operation. The release of these critical medications is often delayed due to the limited testing capacity, sample shipment, and other logistic issues.

Herein, we report an ultrasensitive and universal method for the detection of NDMA in different pharmaceutical products using a novel full evaporation static headspace GC method with nitrogen-phosphorous detection (FE-SHSGC-NPD). The method sensitivity was improved by eliminating the headspace-liquid partition, and the *in situ* nitrosation, a common issue encountered in GC analysis of nitrosamines, was overcome by employing an effective inhibition scheme. The method performance characterization and potential application as a universal method for semi-volatile nitrosamines across different pharmaceutical products were demonstrated.

## EXPERIMENTAL

### Instrument

All studies were performed using an Agilent 7890B gas chromatography system equipped with nitrogen phosphorous detector and a 7697A headspace sampler, and all data were acquired using Waters Empower 3 software.

### Sample Preparation

The experimental conditions for the analysis of NDMA in metformin products are as follows. The experimental conditions for other studies will be specified in the figure caption if they are different. One advantage of FE-SHS is the flexibility in sample size, which could be varied from sub mg to ~100 mg to achieve the desired sensitivity. The sensitivity in ppb is inversely proportional to sample size. The diluent contains 20 mg/mL pyrogallol and 0.1% v/v phosphoric acid in isopropanol. Standard solutions were prepared at 50 μg/mL in isopropanol and then diluted with diluent to 20 ng/mL. Reporting limit (sensitivity) solution was prepared by diluting the standard solution 1:10 to 2 ng/mL. Sample solutions were prepared by grinding the tablet into a fine powder and transferring a portion equivalent to 21 ± 5 mg metformin HCl into a 10 mL headspace vial. A pipette was used to accurately deliver 50 μL of diluent into the headspace vial, which was immediately capped tightly for analysis. Refer to Supplemental Information 1 for details. Please note that nitrosamines are potent probable carcinogens and should be handled carefully according to the safety data sheet (SDS).

### Headspace Parameters

The headspace vial volume was 10 mL. The vial was heated in the headspace oven at 115°C for 15 min with high shaking. The sample loop volume was 1 mL, with the injection loop temperature of 160°C and the transfer line temperature of 170°C. Before injection, the vial was pressurized to 30 psi. The equilibration time was 0.1 min, and the injection time was 0.5 min. Refer to Supplemental Information 2.1 for details.

### GC Parameters

All GC analysis was performed using a G16 column (e.g., Agilent DB-Wax), 30 m × 0.25 mm I.D. and 0.5-μm film thickness. Helium was used as the carrier gas at a constant flow rate of 3 mL/min. The GC inlet had a temperature of 200°C and a split ratio of 5:1. The oven was programed to hold 60°C for 1.5 min, ramp at 20°C/min to 150°C, and then 40°C/min to 240°C and hold for 3 min for a total run time of 11.25 min. A nitrogen phosphorous detector with a BLOS bead was used for sensitive detection of nitrosamines, with a temperature setting of 330°C, hydrogen (fuel) flow of 3 mL/min, air (oxidizer) flow of 60 mL/min, and constant makeup gas (nitrogen or helium) at 5 mL/min. An offset of 20 pA was used and is recommended for future analysis. Refer to Supplemental Information 2.2 for details. Overlaid representative chromatograms are shown in Fig. S1.

## RESULTS AND DISCUSSION

### Improved Sensitivity Using Full Evaporation Static Headspace (FE-SHS) Sampling

Traditional static headspace (SHS) sampling is often not effective for the analysis of nitrosamines as they have relatively high boiling points (e.g., 151°C for NDMA, the smallest nitrosamine) and high partition coefficients in many sample diluents, thus resulting in low sensitivity (19). Inspired by the concepts of multiple headspace extraction (MHE)(20, 21) and the full evaporation technique (FET) (22–24), we propose a new headspace sampling technique for the analysis of nitrosamine in solid dose pharmaceutical products. It is essentially a headspace extraction of solid sample carried out in a single step instead of multi-steps in MHE, which reduces extraction time and improves the extraction efficiency and thus sensitivity. Samples are prepared by grinding tablets into a fine powder and transferring a small aliquot into a headspace GC vial. Nitrosamines are extracted to the headspace by heating at high temperature and analyzed by GC-NPD. The extraction time is dependent on the time it takes for the analytes of interest to diffuse from solid to headspace, which can be shortened by reducing the particle size through grinding and increasing the headspace heating temperature. The former is quite straightforward as most drug products can be easily ground into fine powder using mortar/pestle or mechanical grinder, but the effects of varying heating temperature are more complex. At low temperature, the diffusion is slow, and nitrosamines may adsorb to the solid sample matrix, which reduces method sensitivity. At high temperature, the sample matrix may decompose and interfere with peak of interest. Because the expected level of nitrosamine is extremely low (< 1 ppm), it is easy to drive all nitrosamines to the headspace even at low temperatures. This allows us to achieve sensitive detection of all tested nitrosamines at a headspace oven temperature below their boiling points. To quantitate nitrosamines, a small aliquot of external standard is added to the headspace vial and extracted using the same parameters. The volume of standard solution is small enough that it will be fully evaporated upon heating, akin to that in full evaporation technique (FET) (22–24). The same amount of diluent is added to the headspace vial containing the solid sample.

**Figure 1:**
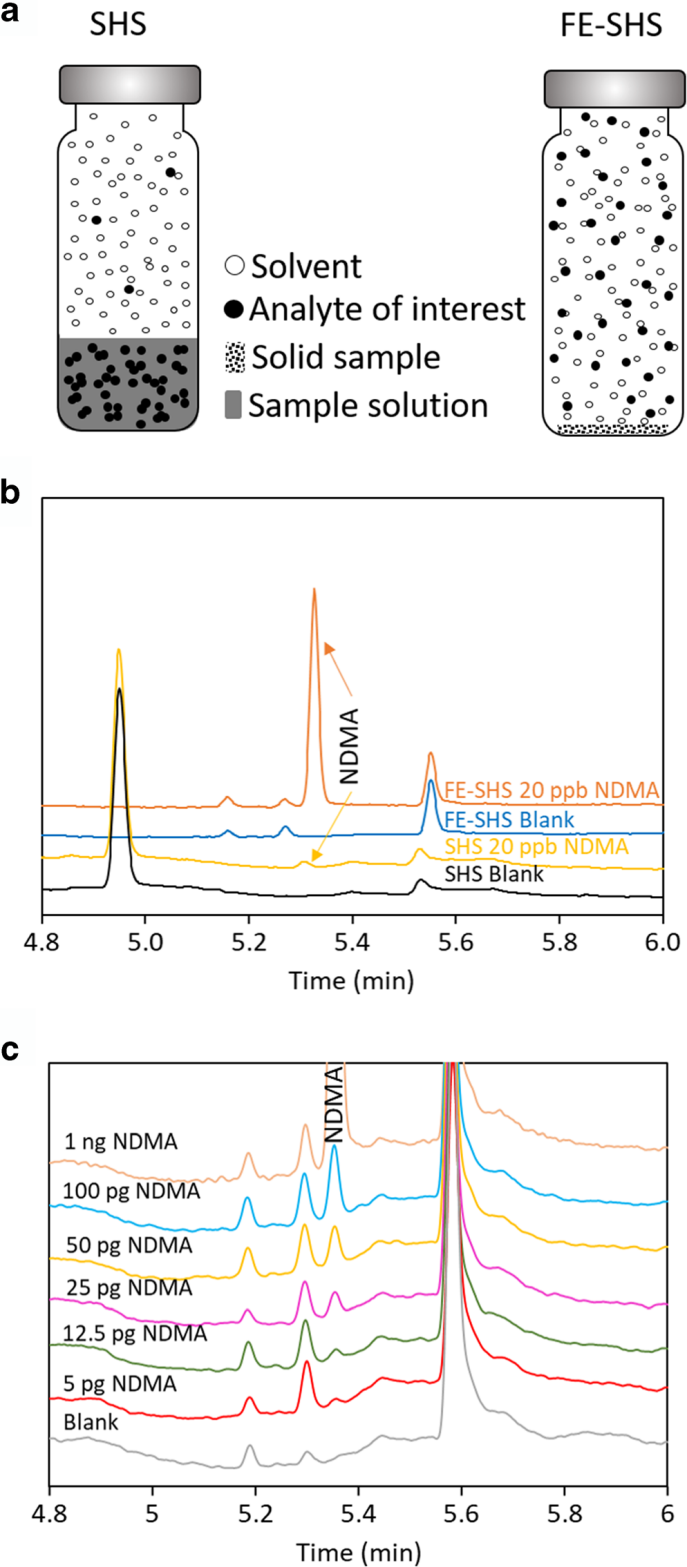
**a** Schematic illustration of traditional static headspace sampling (SHS) and full evaporation static headspace sampling (FE-SHS). **b** Comparison of SHS and FE-SHS for the analysis of NDMA. Experimental conditions are as described except for starting with an oven temperature of 50°C as opposed to 60°C. **c** Overlaid chromatograms of NDMA standard solutions from 5 pg to 1 ng analyzed using FE-SHS-NPD

Fig. 1a shows a schematic comparison of traditional static headspace (SHS) sampling and full evaporation static headspace (FE-SHS) sampling. In traditional SHS, the analyte of interest with high boiling point (e.g., nitrosamine) mainly stays in the liquid phase due to its high partition coefficient, resulting in low sensitivity. In FE-SHS, analyte of interest and the solvent is fully evaporated into headspace, while the sample matrix is not volatilized. Without the undesirable partition, better sensitivity can be achieved even though a smaller amount of sample is used. Fig. 1b shows the comparison of sensitivity for the analysis of NDMA using SHS and FE-SHS. For static headspace sampling, 1 mL of 2 ng/mL NDMA in DMSO was added to a 10 mL headspace vial, which corresponds to 20 ppb NDMA with respect to 1 mL of 100 mg/mL sample. For FE-SHS, 50 μL of 20 ng/mL NDMA in diluent containing 20 mg/mL pyrogallol and 0.1% phosphoric acid in IPA was added to a 10 mL headspace vial, which corresponds to 20 ppb with respect to 50 mg sample.

The sensitivity with FE-SHS is 38 times higher than SHS. The improved sensitivity in FE-SHS can be attributed to the elimination of headspace-liquid partition. In addition, the injection volume for SHS is limited due to “phase soaking” effect of condensed solvent (25, 26), while the injection volume can be easily increased for FE-SHS (e.g., from 1 to 5 mL) to further improve the sensitivity as IPA is volatile and does not exhibit this adverse effect. Fig. 1c shows the overlaid chromatograms of blank and NDMA standard solutions obtained using FE-SHSGC-NPD ranging from 5 pg to 1 ng in a headspace vial, corresponding to 0.1 to 20 ppb with respect to 50 mg sample size. The detection limit (DL) and quantitation limit (QL) are estimated to be 0.1 ppb and 0.25 ppb, based on a signal to noise ratio of 3:1 and 10:1, respectively, which is significantly more sensitive than those obtained using LC-HRMS method (QL: 30 ppb) (27). Refer to Supplemental Information Fig. S2 for the integration and calculation of s/n ratio for QL solutions. The sensitivity obtained using this method readily meets the regulatory requirement, e.g., 10% AI or 4.8 ppb NDMA in metformin HCl based on the AI of 96 ng and a maximum daily dose of 2 g.

### Inhibition of *In Situ* Nitrosation

**Figure 2:**
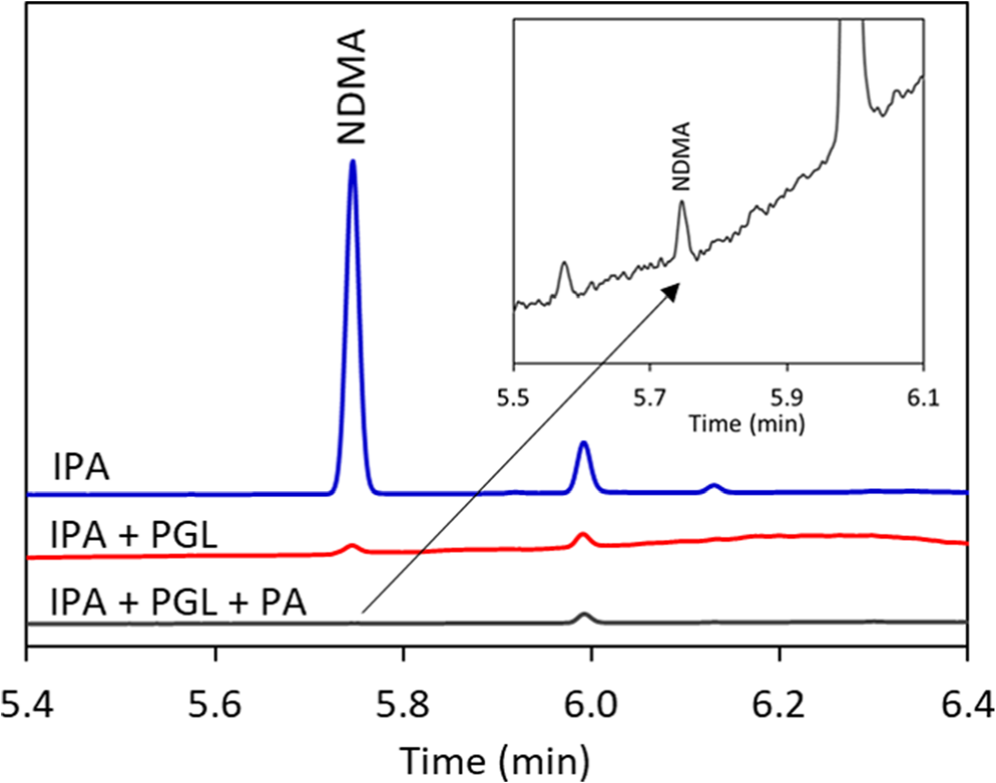
Inhibition of *in situ* formation of NDMA during analysis by FE-SHSGC-NPD. About 30 mg metformin HCl drug substance was added to the headspace vial with 50 μL diluent containing (1) isopropanol (IPA), (2) 20 mg/mL pyrogallol (PGL) in IPA (IPA + PGL), or (3) 20 mg/mL pyrogallol and 0.1% phosphoric acid (PA) in IPA (IPA + PGL + PA)

One common issue during nitrosamine analysis is the *in situ* formation of nitrosamine from secondary amines that may be present in the drug substance with nitrosating agents such as nitrite which are ubiquitous in excipients (28). This is especially of high risk for headspace GC analysis due to extended heating at high temperature. This *in situ* formation of nitrosamine could cause false positives and incorrect rejection of acceptable products (11, 18). In order to quantitate nitrosamine accurately, *in situ* formation of nitrosamine must be eliminated. There have been tremendous efforts in the past to inhibit nitrosation of reactive amines in solution, but to our knowledge, there are a few reports on inhibiting the *in situ* formation of nitrosamine for analytical purposes (28–31). Several scavengers for nitrosating agents including pyrrole, 2,5-dimethylpyrrole, pyrogallol, phloroglucinol, caffeic acid, catechol, ascorbic acid, hydrazine, propyl gallate, and gallic acid have been evaluated for this method and were found to inhibit nitrosation at various levels. A combination of pyrogallol and phosphoric acid in isopropanol solvent was found to provide the best inhibition effect in solid matrix as shown in Fig. 2.

The metformin HCl reagent contains no NDMA based on testing using an LC-MS method (detection limit = 10 ppb) (27). However, when the same material was analyzed using FE-SHS-NPD method, 1000 ppb NDMA was detected using isopropanol as diluent, suggesting significant *in situ* formation. The NDMA level decreased to 28 ppb when 20 mg/mL pyrogallol in IPA was used as diluent, which is still a false positive level due to *in situ* formation. When 20 mg pyrogallol in 0.1% phosphoric acid in IPA was used as diluent, the NDMA level decreased to 0.7 ppb, which is below 10% of AI or 4.8 ppb. We believe that 0.7 ppb NDMA is intrinsic in this material, but this cannot be verified as there is no other method that provides this level of sensitivity. As such, this combination of pyrogallol and phosphoric acid in IPA has effectively inhibited the *in situ* formation of nitrosation resulting from headspace extraction, limiting the potential for false positive. The nitrosation inhibition effect of phenolic compounds including pyrogallol has been reported previously (32–37). However, it is unexpected that the addition of acid further improves the inhibition effect as nitrosation often accelerates under acidic conditions (38). It is possible that dimethylamine, the precursor for NDMA, is fully protonated in the presence of 0.1% phosphoric acid, and the nitrosation proceeds extremely slowly, if at all. On the other hand, pyrogallol in acidic condition reacts readily and irreversibly with nitrosating agents, which are consumed almost completely (39). One benefit of using pyrogallol as nitrosation inhibitor is its low volatility and weak response on NPD, resulting in low baseline noise.

### Method Accuracy

**Figure 3:**
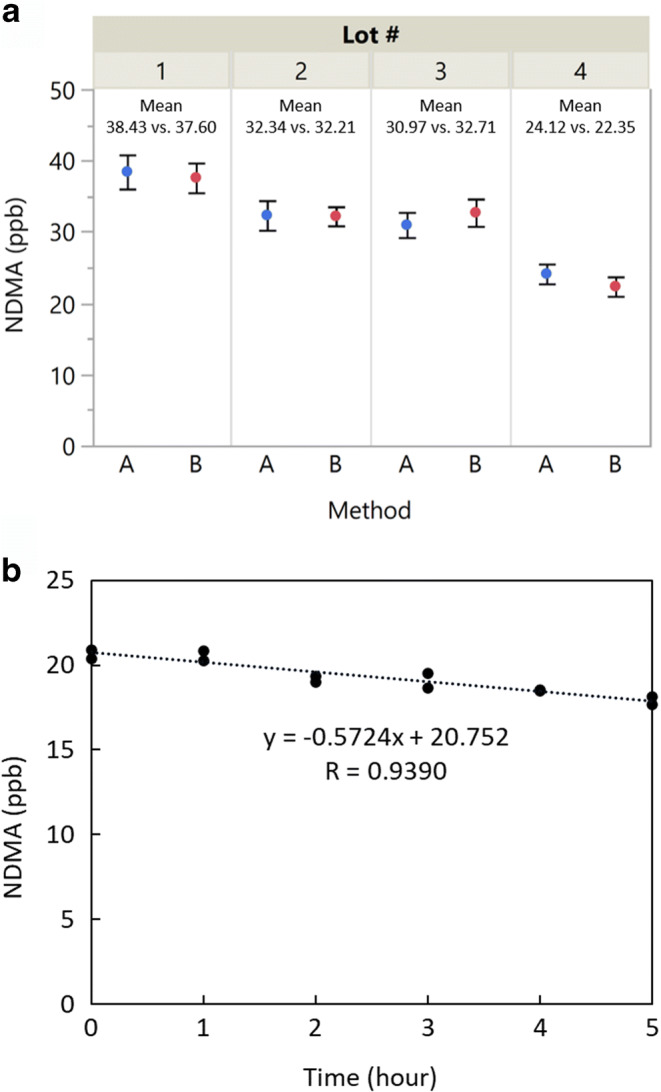
A Comparison of NDMA results using FE-SHSGC-NPD method (**a**) and LC-MS method (**b**). B Loss of NDMA in ground tablet powder across time, held at ambient temperature

To demonstrate method accuracy, four lots of metformin HCl extended release drug products containing high levels of NDMA were analyzed using both a LC-HRMS method (27) and the FE-SHSGC-NPD method. The results were analyzed using JMP software 14.1.0 as shown in Figure 3a. The mean difference between two methods is 0.25 ppb, with a 95% confidence interval of 0.25 ± 2.36 ppb and a *p*-value of 0.7610, suggesting that the difference between these two methods is due to random variations.

For FE-SHSGC-NPD analysis, it is necessary to grind the tablet into fine powder in order to facilitate the extraction of nitrosamines to headspace. The stability of the ground metformin extended release tablet at ambient temperature is shown in Figure 3b. The NDMA level decreased by about 0.57 ppb or 2.8% per hour over the course of 5 h. Although the difference is not considered significant, and may not be noticeable using a less sensitive method, a consistent trend was observed using the FE-SHSGC-NPD method. This demonstrates that the nitrosamines can be driven to headspace at sub-boiling point temperatures, which supports the underline principles of this method. For accurate quantitation, it is necessary to transfer the ground tablet powder into the headspace vial, add diluent, and close the vial as soon as possible.

One common issue for the quantitative MS analysis of nitrosamines is the requirement for the use of internal standard due to several limitations including sample volume expansion at high concentrations, evaporation (e.g., DCM extraction/reconstitution), and variations in ionization efficiency. All these concerns are not applicable to the FE-SHSGC-NPD method due to elimination of sample extraction and lack of ionization of NPD. The %RSD of bracketing standards across a 24-h run is typically <5%, which is much tighter than the corresponding LC/MS and GC/MS method without internal standard and provides significant time and cost savings in QC environment.

### Method Universality

For traditional methods, different sample preparation steps are often required for different drug products to extract nitrosamines from the sample matrix. However, there is no need for sample extraction prior to analysis using this method, making it a potentially universal method to analyze NDMA in different drug substances and drug products as is or with minor modifications. In addition, this sample preparation method is particularly advantageous for samples that are prone to gelling, especially in the presence of water.

**Figure 4:**
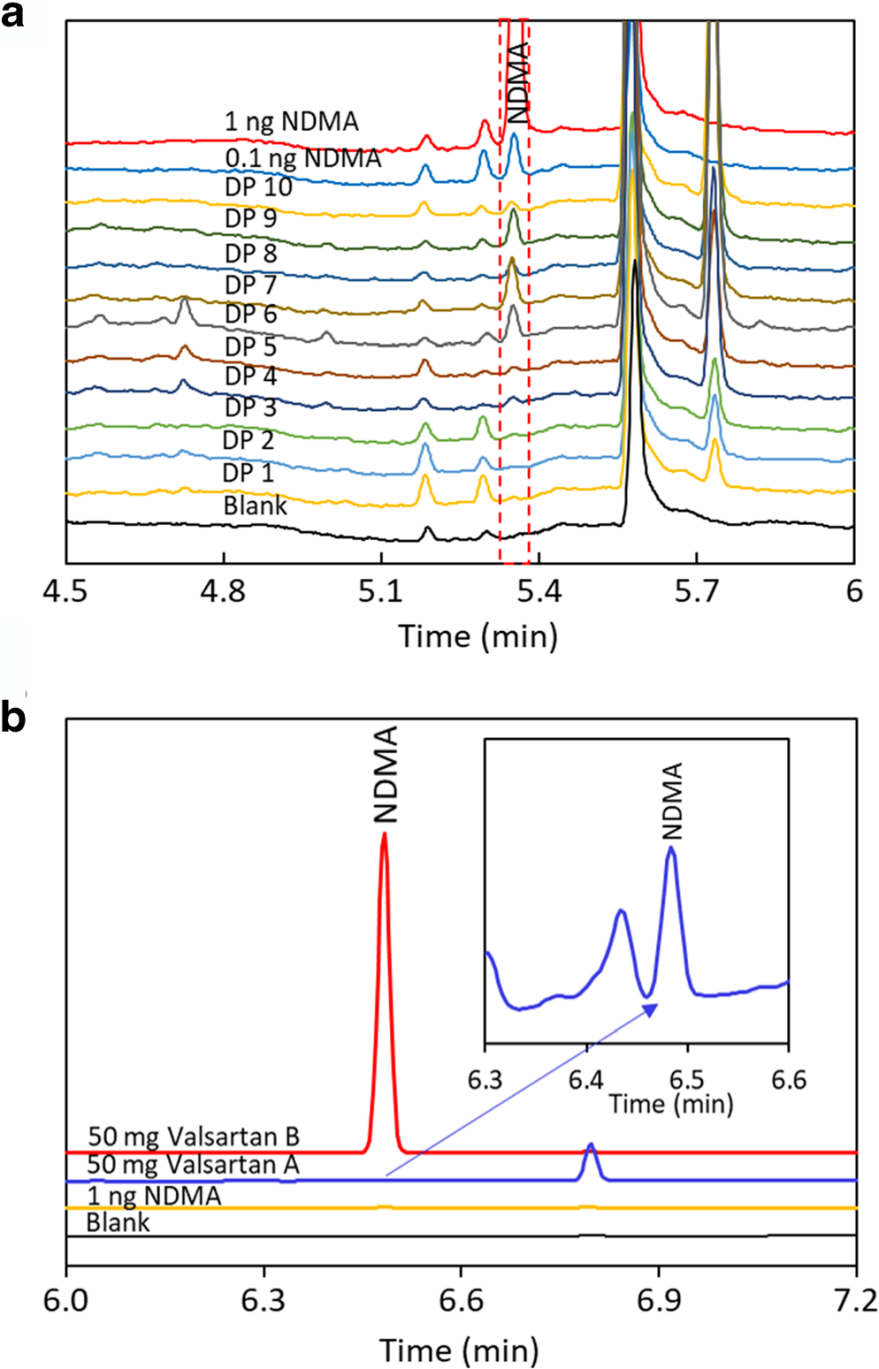
**a** Analysis of 10 different fixed dose combination drug products containing metformin HCl using FE-SHSGC-NPD method. **b** Analysis of NDMA in valsartan drug substances. The oven temperature is held at 50°C for 1.5 min, then ramped by 15°C/min to 150°C, followed by 40°C/min to 240°C, and finally held at 240°C for 3 min. Approximately 50 mg valsartan drug substance was used for analysis

Fig. 4a shows the analysis of NDMA in 10 different drug products containing metformin HCl, and Fig. 4b shows the analysis of NDMA in valsartan drug substances using the same FE-SHSGC-NPD method. The NDMA levels range from <1 to 5 ppb for metformin HCl drug products. The NDMA results are 4 and 6900 ppb for valsartan drug substance lot A and valsartan drug substance lot B, respectively. The same method has also been successfully applied to detect NDMA in multiple drug substances in development stage, in which the risk of NDMA contamination was identified. These results demonstrated the potential universality of this method.

**Figure 5:**
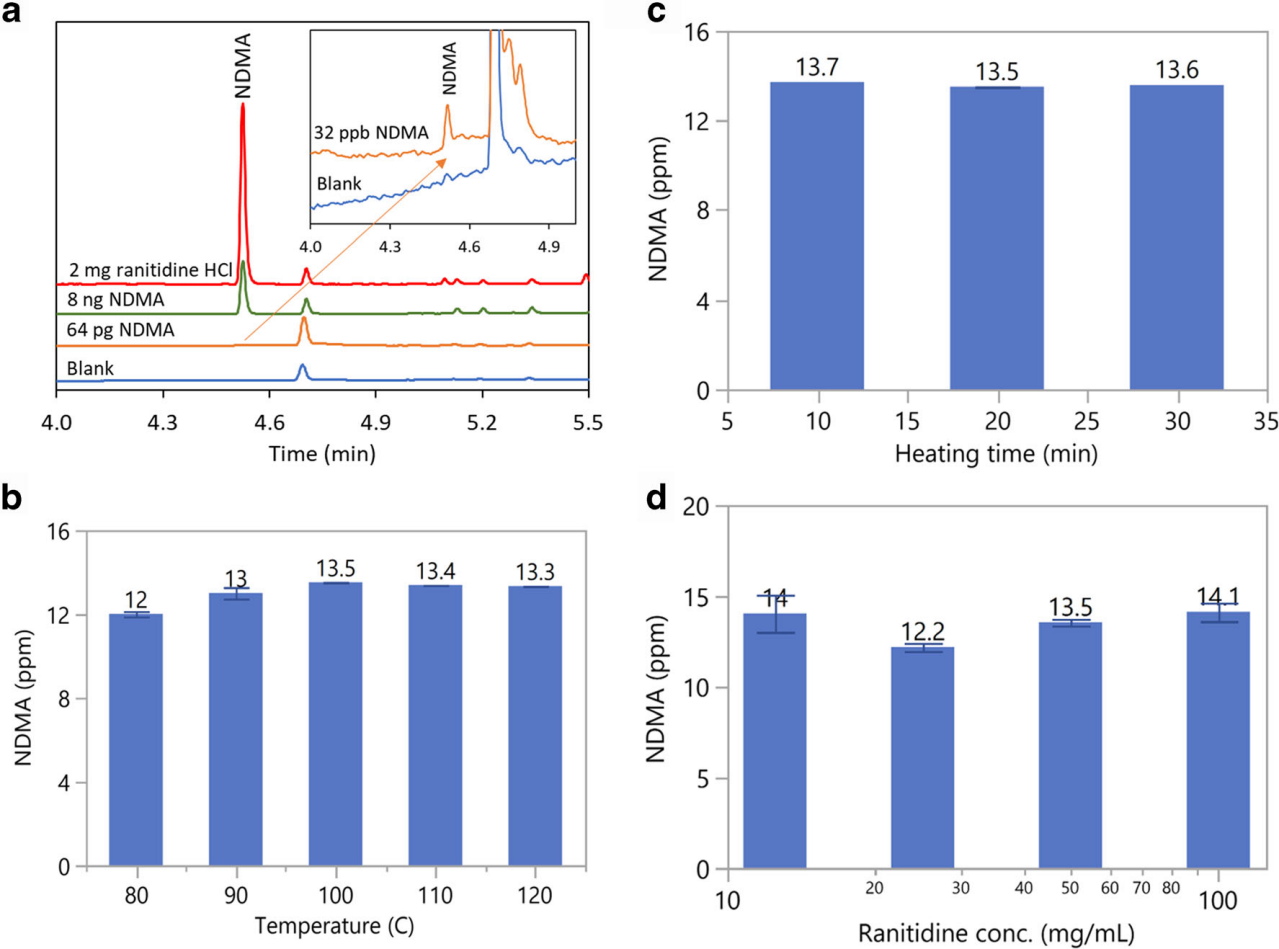
**a** Analysis of NDMA in ranitidine HCl drug substance. The oven temperature is held at 60°C for 1.5 min, then ramped by 30°C/min to 240°C, and finally held at 240°C for 2 min. For sample preparation, 50 mg/mL ranitidine HCl was dissolved in a diluent containing 100 mg/mL pyrogallol, 20 mg/mL diphenylamine, and 0.1% phosphoric acid in methanol. For GC analysis, 40 μL sample solution was transferred into a 20-mL headspace vial. The vial is equilibrated at 100°C for 20 min. **b** Analysis of NDMA in ranitidine HCl using FE-HSGC-NPD with different headspace oven temperatures from 80 to 120°C. **c** Analysis of NDMA in ranitidine HCl using FE-HSGC-NPD with different equilibration times from 10 to 30 min. **d** Analysis of NDMA in ranitidine HCl using FE-HSGC-NPD with different sample concentrations ranging from 12.5 to 100 mg/mL

This method was also applied to detect NDMA in ranitidine HCl with minor modifications. GC is generally considered unsuitable for the analysis of NDMA in ranitidine as it is known that ranitidine degrades at elevated temperature to form NDMA, causing analytical artifact (5, 18). Abe *et al.* reported over 100-fold increase of NDMA during headspace GC-MS analysis when the headspace oven temperature was increased from 80 to 110°C (40). When the method described in Fig. 4a was applied to analyze NDMA in a ranitidine HCl drug substance, about 28.3 to 52.0 ppm NDMA was detected, compared to 13.2 ppm obtained using LC-MS method, indicating analytical artifact due to *in situ* NDMA formation. To accurately quantitate NDMA in ranitidine using FE-HSGC-NPD method, the diluent was modified to include 100 mg/mL pyrogallol, 20 mg/mL diphenylamine, and 0.1% phosphoric acid in methanol, and the ranitidine HCl sample size was reduced from 50 to 2 mg. The increased ratio of pyrogallol to sample size (125×) further improves the scavenging efficiency of nitrosating agents. The addition of diphenylamine ensures that remaining nitrosating agents are completely consumed before they react with residual dimethylamine in ranitidine as diphenylamine is a weaker base (pKa~0.8) and thus a much more reactive nitrosating substrate under acidic conditions (38). The results are shown in Fig. 5a. The method quantitation limit is 32 ppb NDMA with respect to 2 mg ranitidine HCl sample, which is 10% of the AI limit of 320 ppb. The NDMA results in ranitidine HCl drug substance is 13.5 ppm, which matches well with the result of 13.2 ppm obtained using LC-HRMS. The *in situ* formation of NDMA was completely inhibited, evidenced by the consistent NDMA results obtained under varied conditions including (1) varying the headspace oven temperature between 80 and 120°C (Fig. 5b), (2) changing the headspace equilibration time from 10 to 30 min (Fig. 5c), and (3) changing the sample concentration from 12.5 to 100 mg/mL(Fig. 5d). Considering that ranitidine is one of the most difficult compounds for GC analysis, we believe that the results above serve as strong testimonies of the universal applicability of this FE-HSGC-NPD method.

For the results shown in Fig. 5, ranitidine HCl drug substance was dissolved in diluent and pipetted into the headspace vial instead of weighing small quantity of each sample separately (0.5 to 4 mg). Therefore, the sample extraction appears to be similar to FET proposed by Markelov (21). However, in practice, the solid sample can be weighed into the headspace vial with subsequent addition of diluent to dissolve it to avoid additional extraction step, especially for drug product. To minimize analytical variability due to small sample size, the sample should be homogenized thoroughly, and duplicate analyses should be performed.

**Figure 6:**
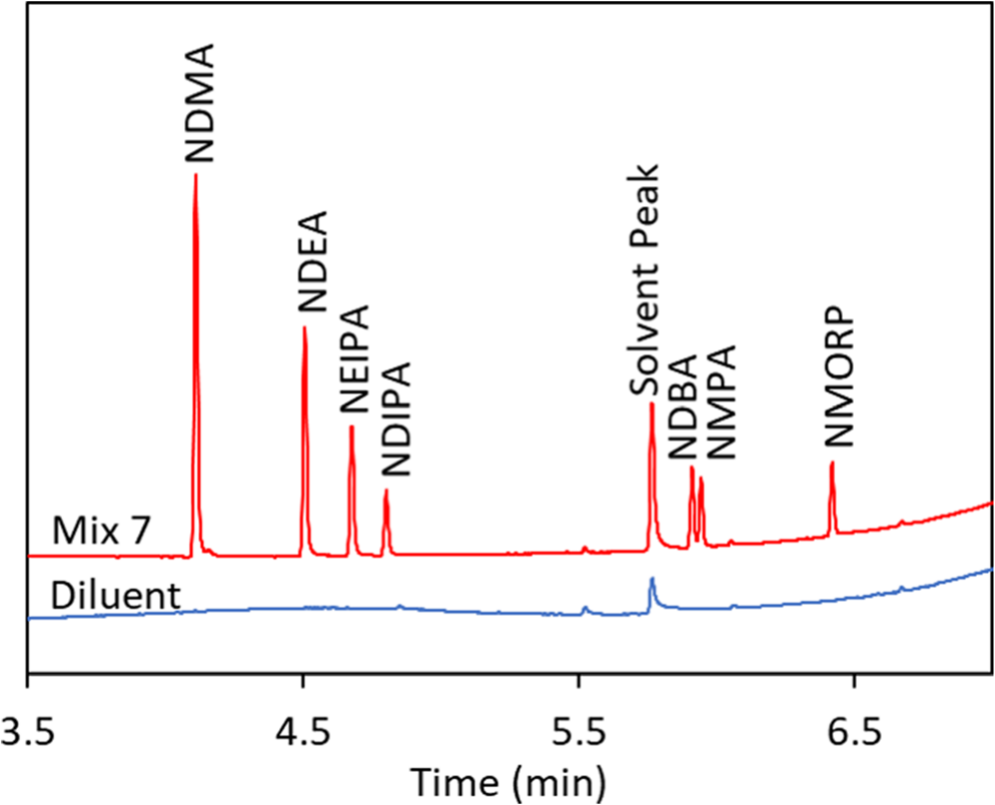
Analysis of seven common nitrosamines including NDMA, NDEA, NEIPA, NDIPA, NDBA, NMPA, and NMORP using FE-SHSGC-NPD. The carrier gas is helium at constant flow rate at 2 mL/min. The oven temperature is held at 70°C for 1 min, then ramped by 25°C/min to 240°C, and finally held at 240°C for 1 min. The diluent contains 20 mg/mL pyrogallol and 0.1% phosphoric acid in IPA. The standard solution contains 500 ng/mL each of NDMA, NDEA, NEIPA, NDIPA, NDBA, NMPA, and NMORP, and 40 μL is added to a 20-mL headspace vial, capped and crimped tightly. The vial is heated at 150°C for 30 min, and 1 mL headspace is injected with a split ratio of 20:1

In addition, this method can be applied to analyze other common nitrosamines with established AIs including N-nitrosodiethylamine (NDEA), N-nitrosoethylisopropylamine (NEIPA), N-nitrosodiisopropylamine (NDIPA), N-nitrosodibutylamine (NDBA), N-nitrosomethylphenylamine (NMPA), and N-nitrosomorpholine(NMORP) as shown in Figure 6. Higher headspace oven temperatures may be required to minimize the adsorption to the solid matrix for nitrosamines with high boiling points.

The method has been validated according to ICH guideline and has been demonstrated to be sensitive, specific, accurate, and precise as shown in the summary of method validation results (Table S1). It has been used successfully to test 795 batches of commercial metformin HCl products in 11 days on one headspace GC instrument including both instant and extended release formulations, demonstrating the capability for high-throughput analysis.

## CONCLUSIONS

In conclusion, a sensitive FE-SHSGC-NPD method was developed to analyze NDMA in pharmaceutical products. This new method boasts simple sample preparation, low-cost instrumentation (same cost as headspace GC-FID), and easy data processing, making it an ideal choice for routine testing at any analytical laboratory. The analytical artifact that is often encountered during nitrosamine analysis is eliminated with efficient inhibition of *in situ* nitrosation. The method has been successfully applied to test for NDMA in different drug products and drug substances including the very difficult ranitidine HCl, demonstrating the potential as a universal method for different pharmaceutical products. Widespread adoption of this technique for nitrosamine analysis could play a key role to ensure patient safety, expedite drug development process, and minimize the interruption to the supply of critical medications. Other application of this technique may include the analysis of trace level impurities with moderate volatility beyond nitrosamines.

## Supplementary information

ESM 1 (DOCX 107 kb)
