# Supplementary material for: A Full Evaporation Static Headspace Gas Chromatography Method with Nitrogen Phosphorous Detection for Ultrasensitive Analysis of Semi-volatile Nitrosamines in Pharmaceutical Products
Source: AAPS J. 2022 Jan 6;24(1):23. doi: 10.1208/s12248-021-00669-8 (PMC8817102; doi:10.1208/s12248-021-00669-8)
Supplement: Supplementary file 1 — (DOCX 107 kb) [file 12248_2021_669_MOESM1_ESM.docx]

Supplementary Materials

# **Materials and Methods**

# List of reagents:

- Pyrogallol: Alfa Aesar, ACS reagent, MA
- Isopropanol: Fisher Chemical, Optima grade, PA
- Methanol: Fisher Chemical, Optima grade, PA
- Phosphoric acid: Fisher Chemical, HPLC grade, PA
- Milli-Q water: Millipore, MA
- Metformin HCl: Alfa Aesar, ACS reagent, MA
- Valsartan: BLD Pharm, OH
- Valsartan: Oakwood Chemical, SC
- Ranitidine HCl: Sigma, MO

# Example Method Parameters:

## Headspace parameters:

| Vial size: | 10 mL |
| --- | --- |
| Vial oven temperature: | 115°C |
| Injection volume (Sample Loop): | 1 mL |
| Loop temperature: | 160°C |
| Transfer line temperature: | 170°C |
| Vial shaking mode: | High |
| GC cycle time: | 14 min |
| Vial equilibration time: | 15 min |
| Pressure equilibration time: | 0.1 min |
| Injection time: | 0.5 min |
| Vial fill mode: | Fill to pressure at 30 psi |

## GC Parameters:

| GC column: | G16, 30 m x 0.25 mm, 0.5 μm film thickness, e.g. Agilent DB-Wax, Catalogue number: 122-7033 or equivalent) |
| --- | --- |
| Inlet Temperature: | 200 °C |
| Split Ratio: | 5 : 1 |
| Carrier Gas: | Helium |
| Flow Rate: | Constant flow at flow rate 3 mL/min |
| Detector: | Nitrogen Phosphorous Detector (NPD)  Temperature: 330 °C  Fuel flow (Hydrogen): 3 mL/min  Oxidizer flow (Air): 60 mL/min  Makeup gas: Nitrogen or Helium, constant makeup at 5 mL/min  Recommended Offset: 20 pA |
| Oven Temperature Program: | Hold 60 °C for 1.5 min, ramp at 20 °C /min to 150 °C, then 40 °C/min to 240 °C and hold for 3 min |
| Total run time: | - 1. in |

Fig. S1. Representative chromatograms of blank, QL, standard and samples.

Fig. S2. Calculation of USP s/n ratio for quantitation limit solution (50 uL of 0.25 ng/mL NDMA or 12.5 pg) in Empower


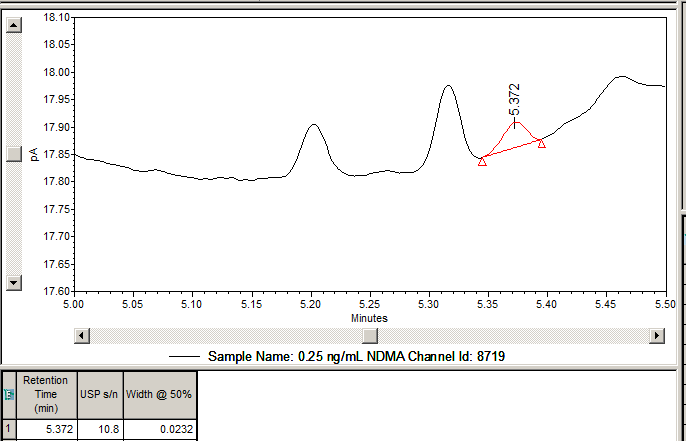


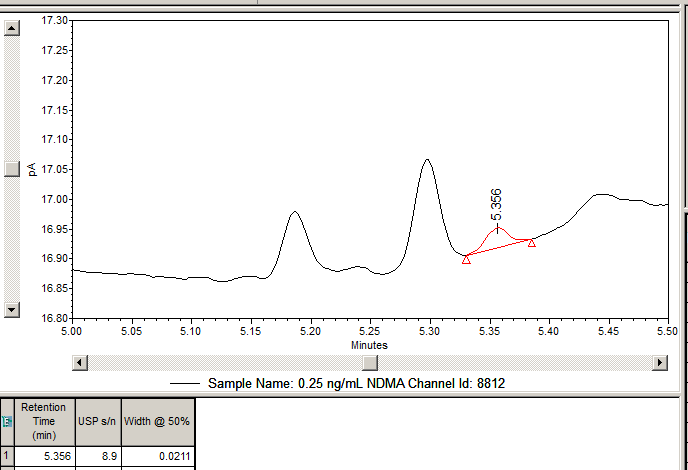


# **Table S1. Summary of Method Validation Results**

| **Validation Characteristic** | **Targeted Acceptance Criteria** | **Validation Results** |
| --- | --- | --- |
| Specificity | No interference from blank injection or sample matrix | Pass |
| Linearity | R > 0.950 | R = 0.999 |
| Accuracy | Mean %Recovery at each concentration level is 75-125% | Mean % Recovery at each concentration level: 85% - 108% |
| Quantitation Limit | S/N ratio ≥ 10 | S/N ratios: 20 – 83 for 4.8 ppb NDMA |
| Detection Limit | S/N ratio ≥ 3 | S/N ratios: 16 – 24 for 2.4 ppb NDMA |
| Measurement Precision | %RSD (n=6) ≤ 25% | %RSD: 4 - 6% |
| Repeatability | %RSD of the %recovery from 9 spiked samples ≤ 30% | %RSD of the %recovery from 9 spiked samples: 4 – 5% |
| Intermediate Precision | %RSD of the %recovery from 9 spiked samples ≤ 30% for each analyst  %RSD for %recovery from 18 pooled results from both analysts ≤ 30%  %Difference between mean %recovery from two analysts ≤ 25% | Analyst 1: %RSD 4 – 5%  Analyst 2: %RSD 4 – 9%  %RSD for %recovery from 18 pooled results from both analysts: 7 – 8%  % Difference between mean %recovery: 6 – 13% |
| Range | Range is demonstrated from reporting Threshold to 120% of specification | Range 4.8 ppb to 96 ppb relative to metformin HCl, which represents 10% to 200% of specification limit of 48 ppb |
| Solution Stability | QL: S/N ratio ≥ 10  % Difference in standard and stock standard solution is within ± 25% | All solutions are stable for up to 8 days at room temperature |
| Ground Sample Stability | % Difference in ground samples at different time point is within ± 25% | Ground samples are stable for up to 6 hours in the grinding vial |
